# Immunisation Using Novel DNA Vaccine Encoding Virus Membrane Fusion Complex and Chemokine Genes Shows High Protection from HSV-2

**DOI:** 10.3390/v14112317

**Published:** 2022-10-22

**Authors:** Ursula A. Gompels, Fernando J. Bravo, Sean Briggs, Shima Ameri, Rhonda D. Cardin, David I. Bernstein

**Affiliations:** 1 Virothera, Milner Therapeutics Institute, Cambridge Biomedical Campus, Cambridge CB4 0WS, UK; 2 Division of Infectious Diseases, Cincinnati Children’s Hospital Medical Center, University of Cincinnati, Cincinnati, OH 45221, USA; 3 Department of Pathobiological Sciences, School of Veterinary Medicine, Louisiana State University, Baton Rouge, LA 70803, USA

**Keywords:** DNA vaccine, HSV-2 vaccine, cell fusion, chemokine, virus latency, neutralising antibodies, virus entry, HSV-2 recurrent disease, gene therapy

## Abstract

Herpes simplex virus 1 and 2 infections cause high unmet disease burdens worldwide. Mainly HSV-2 causes persistent sexually transmitted disease, fatal neonatal disease and increased transmission of HIV/AIDS. Thus, there is an urgent requirement to develop effective vaccines. We developed nucleic acid vaccines encoding a novel virus entry complex stabilising cell membrane fusion, ‘virus-like membranes’, VLM. Two dose intramuscular immunisations using DNA expression plasmids in a guinea pig model gave 100% protection against acute disease and significantly reduced virus replication after virus intravaginal challenge. There was also reduced establishment of latency within the dorsal root ganglia and spinal cord, but recurrent disease and recurrent virus shedding remained. To increase cellular immunity and protect against recurrent disease, cDNA encoding an inhibitor of chemokine receptors on T regulatory cells was added and compared to chemokine CCL5 effects. Immunisation including this novel human chemokine gene, newly defined splice variant from an endogenous virus genome, ‘virokine immune therapeutic’, VIT, protected most guinea pigs from recurrent disease and reduced recurrent virus shedding distinct from a gD protein vaccine similar to that previously evaluated in clinical trials. All DNA vaccines induced significant neutralising antibodies and warrant evaluation for new therapeutic treatments.

## 1. Introduction

Herpes simplex virus, HSV-1 and 2, like all human herpesvirus can establish latent infection that persist leading to life-long recurrences. Disease from recurrences range from mild to severe lesions and can be associated with fatal disseminated infections in neonates or immunocompromised patients [1,2,3]. HSV-2 is also a prominent co-morbidity with HIV/AIDS and recent epidemiological studies show even asymptomatic shedding can significantly increase HIV/AIDS transmission [4]. Worldwide HSV-2 prevalence is 13%, which can increase to over 85% of populations in Sub-Saharan Africa [1]. The overall estimates of HSV-2 exposure contributing to incidence of 37% HIV infections [4]. HSV-1 infections are even more common, 66.6% global prevalence, by 49 years age [1]. Recurrent HSV also cause frequent pathology in immunosuppressed cancer patients [5].

Effective drugs have been developed to treat or prevent recurrent episodes of disease, but they do not prevent future recurrences when drug is discontinued, and infected persons can still transmit. Furthermore, HIV co-morbidities persist [6]. Moreover, the drugs have some toxic side effects with life-long use, and there is increasing drug resistance particularly in the most severely afflicted immunocompromised group, with up to 30% with resistant virus, and most transmissions occurring undetected [7]. Therefore, there is a strong unmet need to develop new preventions or treatments, to address both disease and virus shedding. Control of recurrences are essential, and these are composed of both of recrudescence, the disease lesions, and virus reactivation, the secreted virus. However, vaccination approaches have had only limited success [8], and none are approved for use.

Much of the focus for HSV vaccines has been on using envelope glycoproteins which are targets of neutralising antibody. These include mainly the glycoproteins gD and gB, and often in truncated, membrane-free secreted forms which can be purified as recombinantly produced protein. Clinical trial of vaccination using truncated gD and gB proteins with MF59 adjuvant did not protect against HSV-2 [9]. When this truncated gD was formulated with adjuvants monophosphoryl lipid A (MPL) plus alum, termed ASO4, and tested in discordant couples there was 73–74% protection against genital disease in previously HSV seronegative women [10]. However, a subsequent broader phase III study in seronegative women in general, showed no protection to HSV-2, but 58% protection to HSV-1 [8]. In this trial antibodies were shown as a correlate of protection to HSV-1 and weakly to CD4 T lymphocytes [11,12]. The protection seen in the discordant couples suggests the importance of innate factors. Therefore, more complete antigens are required as well as induction of appropriate innate responses which could improve cellular immunity [2], known to control latent infections. To address these issues, we focused on developing DNA vaccines that express the natural conformation of the antigen as well as mediating innate responses to drive cellular immunity.

It is well established that the virus entry complex in the membrane direct binding and cellular fusion. The minimal requirements for in vitro cellular assays for cellular fusion, include a binding component, gD in HSV, and the conserved fusion complex of the fusogen gB, together with the fusion regulator, the gH/gL complex, interacting sequentially to mediate cell fusion [13,14]. Therefore, we recreated the complex expressed in the membrane using DNA immunisation. These form sequential transient interactions after gD binding to its cell receptor, resulting in conformational change in gB, which then can mediate cell membrane fusion. We also evaluated gB SNPs encoding modified gB to stabilise the fusion conformation, and tested this formulation within the encoded virus entry complex without external lipid adjuvants initially, as the complex was already presented in natural virus-like formation in a lipid membrane and utilised the significant advantages of DNA vaccines.

Immunisation using nucleic acid provides significant advantages in induction of cellular immunity [15]. The gene is introduced into the cell, RNA is transcribed in the nucleus, then translated by ribosomes and can be processed by the proteasome for presentation with MHC class I or class II to induce cellular immunity [15]. DNA have the added advantage of thermostability, with the closed circular DNA molecules of plasmid DNA showing particular stability, since nucleases require free ends of DNA for activity. However, adjuvant formulations have been required to increase immune responses, or electroporation to increase amounts of DNA delivered [15].

Expressing the translated glycoproteins within the lipid membrane can increase innate immune responses as membrane fusion is detected by innate sensors through multiple mechanisms. For example, individual fusion mediating glycoproteins interacting with integrin can be sensed by TLR2 [16,17]. While in vivo the membrane fusion event itself can stimulate innate immunity as through cell damage sensing via the cGAS-STING, TLR7 or TLR9 innate sensing pathways compared to only TLR4 for LPS or MPL/GLA derivatives [18,19]. Moreover, immunising HSV genes naturally have high relative CpG composition, which can be further sensed by TLR9.

To further improve DNA vaccines immunogenicity, a direct immune modulator was additionally evaluated. DNA immunisation can be effective for inducing cellular immunity as the pathogen gene is expressed endogenously and presented via Class I MHC to CD4+ or CD8+ T cells. In order to enhance this process chemoattraction for specific cellular subsets were investigated. Earlier studies had combined chemokine genes, such as CCL5, CCL19 or CCL28 with gD DNA vaccines which improved responses in mice, but had not been tested in the guinea pig model for HSV-2, which can evaluate effects on recurrences [20,21]. CCL5 through interaction with its receptor, CCR5, can direct attraction of both activated conventional T lymphocytes as well as T regulator subsets, T-Reg. To inhibit T-Regs and thereby increase transient immune stimulation, inhibition of chemotaxis of T-Regs to a site of immunisation was also considered here. In addition to CCR5, T-Regs express the chemokine receptors CCR4, CCR6 and CCR8 [22,23,24,25]. Small molecule inhibitors of CCR4 or CCR8 expressed on T-Regs subsets have been shown to increase vaccine responses [26,27]. Virus encoded chemokines, can stimulate all these chemokine receptors, as shown for the CC chemokine U83A in HHV-6A, also encoded as a human homologue in the integrated virus endogenous genome [28,29,30]. Therefore, we evaluate here a novel spliced variant, of this human homologue chemokine gene, termed here VIT, virokine immune therapeutic, which retained the binding domain to these receptors, but removed the original signaling domain.

These new approaches to DNA immunisation were tested here in comparison to the original clinically trialed adjuvanted gD protein vaccine in the guinea pig model of HSV-2 infection, which can assess both acute and recurrent disease as well as recurrent virus shedding.

## 2. Materials and Methods

### 2.1. Vaccines

The HSV-2 subunit protein vaccine was composed of truncated gD2 (306) as formulated by G. Cohen (University of Pennsylvania) from Sf9 (*Spodoptera frugiperda*) cells (GIBCO BRL), [31]. The gD2 protein, 5 micrograms, was mixed with 50 micrograms adjuvant MPL (Sigma-Aldrich L6895) made as an aqueous formulation [32] and stored at 4C until addition to the gD2 protein and adsorbed to 500 micrograms aluminium hydroxide, Alhydrogel (Accurate Chemical & Scientific, Carle Place, NY, USA), as described previously [33]. The gD2 protein was also formulated with a proprietary human chemokine formulation CIR, including CCL5, CCL17 and CCL20 (GMP grade, Peprotech/Thermofisher, Princeton, NJ, USA) in sterile saline solution.

The DNA immunisations included formulations of HSV-2 glycoprotein genes encoding gD, gB, gH and gL from HSV-2 strain HG52 (accession numbers gB, UL27, NC_001798.2:c56152-53438 gH, UL22, NC_001798.2:c46570-44054 gL, UL1, NC_001798.2:9463-10137, gD, US6, NC_001798.2:141016-142197), with gB modified to include the HHV-6A culture adapted SNP [34] here designed for HSV-2 gB encoding Thr262Ala, or for a prefusion gB conformation designed here for HSV-2 gB encoding His513Pro, cDNA of the human CCL5 gene (accession BC008600.1), and novel cDNA of spliced variant human chemokine VIT (human genome integrated HHV-6A iciU83A) as identified here by comparisons to the full length long iciU83A gene from the integrated genome [35] (Figures S1 and S2). All genes included their cognate 6bp Kozak sequence, were synthesized (endotoxin free, Blue Heron Biotech/Eurofins, Bothell, DC, USA), sequence verified and expressed from plasmid pCMV6neo containing human CMV transcriptional 5′ enhancer, promoter and start site plus SV40 3′ polyadenylation site (Origene Technologies Inc., Rockville, MD, USA).

### 2.2. Animals and Virus

Female Hartley guinea pigs (Charles River Laboratories, Wilmington, NC, USA), pathogen free, were used (250–350 g), and housed following conditions as approved by the American Association for the Accreditation of Laboratory Animal Care and approved by the Cincinnati Children’s Hospital Research Foundation Animal Care and Use Committee.

Challenge virus used HSV-2 strain MS (ATCC-VR540) [36] which was propagated primary rabbit kidney cells at low passage with subsequent titration on rabbit kidney cell monolayers, as described [37].

### 2.3. Immunisation

A total of 72 guinea pigs were randomised in six cohorts of *n* = 12/group as follows: group 1, no vaccine, group 2, 5 micrograms gD2 protein + MPL/Alum, group 3, 5 micrograms gD2 protein + 5 micrograms CIR protein, group 4 gD2-VLM DNA, group 5 gD2-VLM+CCL5 DNA, group 6 gD2-VLM+VIT DNA.

All DNA immunisations contained 50 micrograms per plasmid DNA (VLM: gD, gB modified, gH, gL, HSV-2 strain HG52; VIT: spliced variant iciU83A) and were formulated in 0.25% bupivacaine as described [38]. All immunisations were injected intramuscularly, i.m., followed by an identical boost after 3 weeks interval.

### 2.4. Virus Challenge

A day before the virus challenge, the animals were bled and sera stored at −20 °C. Three weeks after the second immunisation, animals were challenged with virus intravaginally, i.vag., with 10^6^ plaque forming units, pfu, as described [39]. Cervicovaginal secretions were collected by swabs on days 1, 2, 3, 8 post-inoculation (PI) then stored for assay of virus PFU on Vero cultivated in cGMP BME (Gibco/Thermo Fisher Scientific, Waltham, MA, USA) and 10% FBS (Hyclone, Thermo Fisher Scientific, Waltham, MA, USA).

Each guinea pig was examined daily and scoring conducted for primary genital skin disease. The scoring scale was from 0 to 4, with 0 indicating no disease, 1 for reddening or swelling, 2 for one to three small vesicles, 3 for more than three vesicles, large merging lesions and 4 for several large ulcers with maceration [39,40]. Animals were also evaluated daily from days 14–63 post virus challenge to detect any recurrent herpetic lesions. Recurrent shedding of virus was determined by vaginally swabbing animals three times per week. The swabs were stored at −80 °C until processed for PCR analyses as a marker for virus shedding. At the completion of the study, the guinea pigs were sacrificed, and both dorsal root ganglia (DRG) and spinal cords removed aseptically, and stored at −80 °C prior to DNA extraction for evaluating by PCR for evidence of latent virus infection.

### 2.5. Neutralising Antibody Assay

In brief, sera samples obtained 3 weeks after the last vaccine dose, were heat inactivated, serially diluted two-fold (1:4–1:2048) in media containing 10% rabbit complement (Cedarlane, Burlington, NC, USA) and then mixed with HSV-2 (50–100 pfu) and incubated for 1 h at 37 °C. The samples were then added to 24-well plates seeded with Vero monolayers, and incubated at 37 °C for 1 h followed by a 1.5% methylcellulose overlay. After 3 days at 37 °C, the overlay was removed and plaques enumerated after staining with Crystal Violet (Sigma-Aldrich, St. Louis, MO, USA). The highest dilution producing a ≥50% reduction in plaques was considered the neutralizing antibody endpoint.

### 2.6. Quantification of Virus DNA by PCR

DNA quantification by PCR was performed on vaginal swabs, DRG and spinal cord DNA. The DNA extractions were conducted using QIAamp DNA mini kit (Qiagen, Germantown, TN, USA) according to the manufacturer’s protocol, and the gG2 gene was amplified by PCR using two sets of primers and fam-tamra labelled probe, as described [40,41]. HSV-2 genomic DNA as a positive control was serially diluted into uninfected tissue DNA for determining the genome copy number limit of detection.

### 2.7. DNA Cell Transfection

Human HEK293 cells (National Institute Biologics and Control, London, UK) were cultured in DMEM with 4.5 g/L D-Glucose, L-Glutamine & Pyruvate) (Gibco), 10% FBS, heat inactivated, Australia (Gibco), and 1% Penicillin-Streptomycin (10,000 U/mL) (Gibco). Cells were split and seeded using Trypsin-EDTA (0.05%) (Gibco), 5 min at 37 °C, 5% CO_2_. The 70% confluent cultures were transfected in 12 well plates with cover slips. The 1–2.5 µg DNA, 3 µL Lipofectamine 3000 (Invitrogen, Thermo Fisher scientific, Waltham, MA, USA) and 2–5 µL P3000 (2 µL/µg) (Invitrogen) reagent were added to the media per well. Formulations included no DNA, EGFP plasmid or EGFP plasmid plus VLM, VLM plus CCL5 or VLM plus VIT. Cells were incubated at 37 °C, 5% CO_2_ for 24–72 h then duplicates prepared for processing RNA or Fluorescence. Equivalent transfection efficiencies were followed EGFP fluorescence using an IncuCyte (Sartorius, Göttingen, Germany) 6–72 h.

### 2.8. RNA Purification

Total RNA was extracted 48 h–78 h post DNA plasmid transfection of HEK293 cells using PureLink RNA Mini Kit (Invitrogen) via the manufacturer’s protocol for monolayer cells. Briefly, media was removed from cells, washed, then Lysis buffer (300 µL or 600 µL for < or > million cells) with 10% 2-mercaptoethanol (Invitrogen) added and incubated for 1 min. Cell lysate was removed to a homogenizer with collection tube and centrifuged at 13,000 rpm for 2 min. The flow through was mixed with an equal volume 70% ethanol then centrifuged through a spin cartridge for 15 s. This flow through discarded, followed by one wash cycles with 700 µL wash buffer I followed by centrifugation for 15 s, then two wash cycles with 500 µL buffer II, then the columns centrifuged 2 min to dry the column prior to transfer to an elution tube. Then, 30 µL RNase free water was used to elute the RNA after 1 min incubation at room temperature for 1 min followed by 2 min centrifugation. RNA was used directly or stored at −80 °C.

### 2.9. Reverse Transcriptase Polymerase Chain Reaction Amplification, RT-PCR

All RNA samples were first treated to remove residual DNA using DNaseI Amplification Grade (Invitrogen). The 10 µL reactions included 1 µg RNA sample, 1 µL 10× DNase I reaction buffer, 1 µL DNase I, Amp Garde 1 U/µL within DEPC-treated water or RNase free water and incubated for 15 min at room temperature in one or two cycles of treatment, followed by DNase I was inactivation by addition of 1 µL of 25 mM EDTA and further incubation at 65 °C for 10 min. RT-PCR used Superscript IV One-Step RT-PCR, (Invitrogen) in 25 µL reactions containing 12.5 µL 2× Platinum SuperFi RT-PCR MM (Invitrogen) with 1 µL each of gene specific forward and reverse oligonucleotide primers and 0.25 µL RT enzyme plus 9.25 µL nuclease-free water (Invitrogen) then mixed with 1 µL of DNase treated RNA.

RT-PCR amplification used a reverse transcription step at 50 °C for 10 min, followed by 2 min Hot-Start step at 98 °C followed by 35 cycles of at 98 °C for 10 min, 60 °C for 10 s, and 72 °C for 1 min. Upon cycling completion, extended at 72 °C for 5 min then products separated by 1% agarose gel electrophoresis.

Oligonucleotide primers used in the analyses were for the VIT unspliced and spliced products VTL101-F 5′CTTGTCGAAATGTCCATTCGG3′ and VTL101-R 5′ ATCACACATCGAGCCCTGCTC3′, VTL102-F 5′AAGCTTGTCGAAATGTCCATTCGG 3′ and VTL102-R 5′ GATCATGATTCTTTGTCTAATTTC 3′. For HSV-2 gD, VTL1011-F 5′CTTCACGGCATGGGGCGTTTG3′, VTL1011-R 5′GACTAGTAAAACAATGGCTGG3′. For HSV-2 gH, VTL1013-F 5′ TCACGACCATGGGCCCCGGTC 3′ and VTL1013-R 5′CTAGATTATTCGCGTCTCCAC3′. HSV-2 gL, VTL1014-F 5′CTTCTCGTATGGGGTTCGTC3′ and VTL1014-R 5′ ACTAGTTGCGTCGGAGGCGAG 3′. For CCL5, VTL1015-F 5′ CTTGGTACCATGAAGGTCTCC 3′ and VTL1015-R 5′ GACTAGCTCATCTCCAAAGAG 3′. For VIT, VTL1016-F 5′CTTGTCGAAATGTCCATTCGG3′ and VTL1016-R 5′ATCACACATCGAGCCCTGCTC 3′. For HSV-2 gB, partial gene, iVTL1012-F 5′AGCTTCCCGCCATGCGCGGGGGGG3′, iVTL1012-R5′TGGCATCGGCGTTCTCGACCTTG 3′.

### 2.10. Cell Fusion Assay and Imaging

DNA expression plasmids were transfected into HEK293 cells cultured on glass coverslips. The 24–48 h post transfection coverslips were washed with DPBS (Gibco), then removed and replaced with Live cell imaging solution (Life Technologies, Carlsbad, CA, USA) and inverted on a microscope slide. EGFP fluorescing cells were visualised using ocular 10× and objective 20× (aperture 0.5) magnification on a Nikon Eclipse E600 fluorescent microscope with FITC filter (Excitation 465–495, DM 505, BA 515-55), while Hoechst 3342 (Thermo Fisher Scientific) staining was viewed with UAV-2A filter (excitation 330–380 nm, DM 400, BA420). Hoechst staining was performed prior to viewing by washing cells in DPBS followed by incubation for 10 min at room temperature with 1 microgram/mL Hoechst solution in DPBS. EGFP fluorescing cells images were coded and scaled 1.5× for maximal diameter measurement using Jruler (v1.5, Spadix software and Jimage, Bethesda, MD, USA) set in cm (pixels per inch = 96) with 250–1000 cell counts/5 views. All assays were performed in quadruplicate.

Combined brightfield and two channel fluorescence images were acquired using a ZEISS Axio.VertA1 microscope, (ocular magnification: 1×, objective magnification: 20×, aperture: 0.5, filters: BF, GFP, DAPI, for Hoechst) attached to a ZEISS Axiocam 202 mono microscope camera, using Zen Blue v3.5 software, Cambridge, UK; Oberkochen, Germany, with input microscope magnification for each image captured in black and white, as CZI and TIF files with overlay images constructed and colours applied for each wavelength collected directly imaging the plates. Whole images were analysed to extract fluorescent cell length, using ImageJ 2.1.0 (FIJI) or Zen 3.5 (ZEN Desk). For FIJI analysis, images of EGFP fluorescent cells in CZI format with microscope scaling include measure using the straight-line tool then input to the ROI manager for group measurement and lengths (in um) extracted. Counting was confirmed using the automated Zen software, with the. CZI files set to detect GFP using whole page Frame, automatic and interactive segmentation to ensure cell selection and features set to record ID and Feret maximum. For counting nuclei in EGFP fused cells, a 20-micron threshold was used (excluding 2 dividing cells), then UVA fluorescing nuclei counted.

### 2.11. Statistics

The analyses plan included the following efficacy endpoints: incidence and severity of acute disease, plus effects on virus vaginal replication, recurrent disease, symptomatic and asymptomatic virus shedding, and latent viral burden. Limit on detection were marked and measured for virus quantification at 0.7 log pfu/mL and for qPCR undetectable below the limit of detection at 0.5 log µg copies DNA/mL.

Statistics were performed using Graphpad Prism with one-way Anova using Tukey’s test for multiple comparisons of the means data for the different vaccine treatments vs. no vaccine. Where non-gaussian distributions, non-parametric comparisons using Wilcoxon test and for multiple comparisons Kruskal–Wallis with Dunn’s test. Fisher’s exact test was used for incidence data, with two tailed comparisons. Significance was noted at the *p* values of <0.05 (*), <0.01 (**), <0.001 (***), <0.0001(****).

## 3. Results

### 3.1. Functional In Vitro Gene Expression of the Virus Entry Complex Modulates Cell Fusion

**Figure 1:**
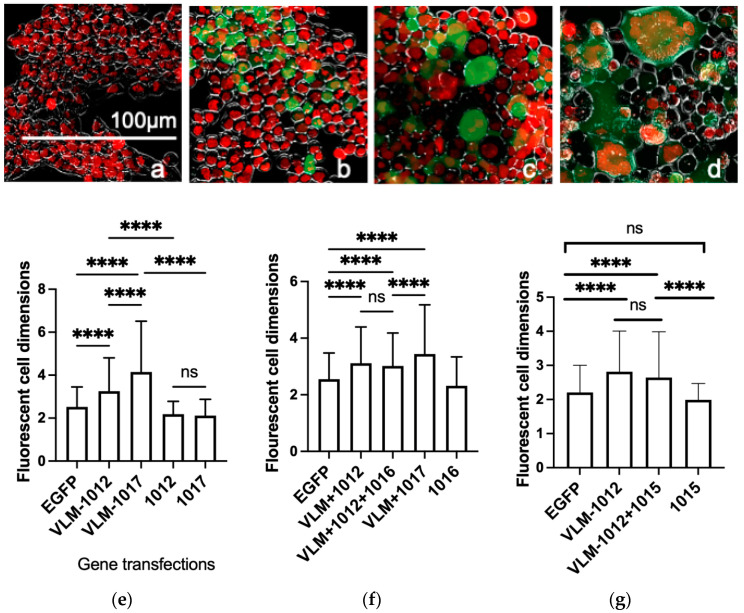
Cell fusion with VLM and VIT gene expression in human cells. VLM gene formulations were examined for effects on HEK 293 cell fusion by transfecting genes together with EGFP genes individually or in combined formulations as viewed for FITC filter fluorescence to show EGFP transfected gene expression, assayed 48–72 h post transfection shown in comparisons to Hoechst live stained nuclei viewed in UVA filter (coloured red to contrast green EGFP), image overlays with brightfield view (**a**–**d**). (**a**) HEK293 cells transfected PBS, (**b**–**d**) with DNA, (**b**) EGFP, (**c**) with EGFP plus VLM of gD (plasmid 1011), gBmut1 (1012), gH (1013), gL (1014), (**d**) with EGFP plus VLM of gD (1011), gBmut2 (1017), gH (1013), gL (1014). (**e**–**g**) The relative cell dimensions where 2 = 10 microns. (**e**,**f**) Mean cell dimensions of EGFP expressing cells comparing effects of 1012 (gB-mut1) to 1017 (gB-mut2) individually or together with gD VLM DNA formulations. (**f**) Comparison of effects 1016 (VIT) DNA addition to gD VLM-1012 formulation and to the gD VLM-1017 formulation and in (**g**) addition of 1015 (CCL5) DNA. (**e**–**g**) transfections measured dimensions of 250–1000 cells in 5 views and representative of 4 independent assays, analysed here by one way ANOVA with Tukey’s test for multiple comparisons, showing mean and standard deviation. *p* < 0.0001 (****) and ns = non-significant above *p* = 0.05.

All the DNA expression plasmids were tested first for expression in cells in vitro prior to immunisation. All DNA plasmids showed positive expression in human HEK293 cells as demonstrated by RT-PCR (Figure S3). In order to determine functional activity of the antigen genes gD, gB, gH and gL, a cellular fusion assay was performed. The EGFP gene was transfected singly or together with the mix of the virus entry glycoprotein genes, gD together with fusion regulators gH and gL and the fusogen gB. Expressed together these should form a virus like membrane, VLM, which is competent to cause cell fusion [13,42,43,44]. In order to stabilise the fusion complex, mutations that stabilise gB fusion conformation were also evaluated. Firstly, the HSV-2 gB gene including the fusion trimer stabilising SNP mutation Thr262Ala in domain I (plasmid 1012), was designed and synthesised here based on tissue culture adapted HHV-6A gB [34]. Effects on stabilising cell fusion were compared to that of gB SNP (plasmid 1017), His513Pro, in the hinge region of gB Domain III, which has been shown to maintain the prefusion conformation by inhibiting conformational changes to the post-fusion state [45]. No transfection and EGFP transfected cells showed single cells (Figure 1a,b). Comparing co-transfection of the VLM mix with novel Domain I or III SNP mutated gB(1012 or 1017), showed more controlled cell fusion with Domain I SNP gB (1012) compared to Domain III SNP gB(1017), maximum 4 compared to 14 nuclei in fused cells, respectively (Figure 1c,d).

Comparisons between the fusion effects were then further evaluated by measuring all the fluorescing cell dimensions (1000 per view) to compare the effects of the two gB fusion stabilising mutations. The results show that within the VLM mix, the gB Domain I mutation, plasmid 1012, mediates significantly less cell fusion than gB Domain III mutation, plasmid 1017, and both gB constructs do not mediate cell fusion in the absence of the total VLM mix with gD, gH and gL as known for wild type virus (Figure 1e,f). The cell fusion was unaffected by co-transfection of the VLM mix with the chemokine molecules, CCL5 or VIT (plasmids 1015 and 1016) (Figure 1f,g). Since gB SNP (plasmid 1017) is a prefusion conformation, but gB SNP (plasmid1012), Thr262Ala showed more controlled cell fusion within the VLM mix, this gB mutation, Domain I (plasmid 1012) was selected for in vivo immunisations of the multivalent VLM formulation with gD, gH and gL.

### 3.2. Gene Immunisation In Vivo Induces Efficient HSV-2 Neutralising Antibodies

**Figure 2:**
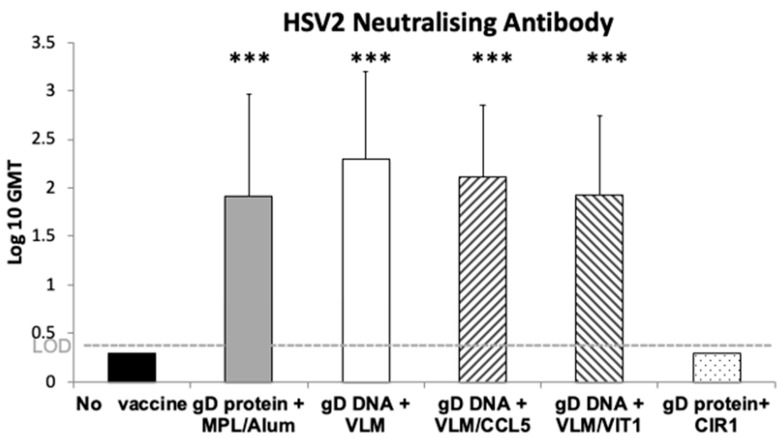
Antibody induction with VLM and VIT genes in guinea pig model. The guinea pigs, 12 per group, were immunised with saline or with the VLM gene formulations on their own or with chemokine CCL5 or VIT compared to positive control gD protein with MPL,3-O-desacyl-4′-monophosphoryl lipid A, with alum adjuvant or gD protein. An additional control immunisation included gD protein with cytokine immune regulator, CIR, human chemokine formulations, agonists with the same chemokine receptor specificity as VIT antagonist, as described in the methods. Guinea pigs were immunised intra muscularly two times separated by three weeks, then sera collected three weeks after the final immunisation. Virus neutralisation assays were conducted and mean end point titers are shown with standard deviation analysed by one way ANOVA with Tukey’s test for multiple comparisons, *p* < 0.001 (***).

Expression as immunogens in vivo was next evaluated using the guinea pig model of HSV-2 infection [39,40]. The VLM formulation used here contained the gB Domain I mutation, plasmid 1012, with gD, gH and gL. The guinea pigs were immunised twice with the DNA formulations, gD DNA within VLM, VLM+VIT or VLM+CCL5 formulations and compared to control formulations: the secreted gD protein with adjuvant MPL/alum as positive control, no vaccine as negative control, and the secreted gD protein plus agonist human chemokine mixtures, CIR, cytokine immune regulators, specific for the same chemokine receptors as VIT. The positive control gD protein vaccine induced significant levels of neutralising antibodies compared to the no vaccine control, while all the DNA vaccine formulations induced antibody levels similar to the gD MPL/alum control immunisation. Immunisation of the gD protein combined with the chemokine agonists of chemokine receptors on T-reg cells (CIR) totally blocked induction of neutralising antibodies, while the antagonist VIT formulation effectively induced them (Figure 2).

### 3.3. Gene Immunisation Highly Protective against HSV-2 Acute Disease and Infection

**Figure 3:**
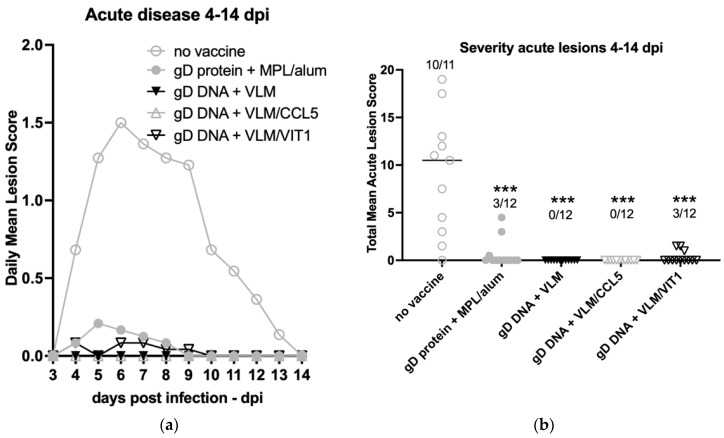
DNA immunisation with VLM vaccines protect against acute disease. Three weeks after the second immunisation, the guinea pigs were challenged with 1 × 10^6^ pfu of HSV-2 (MS strain) and the severity of acute disease (0–14 dpi) were scored. No vaccine (saline) and gD protein + MPL were included as negative and positive controls, respectively. (**a**) The score scale for disease severity ranged from 0 to 4 and the total mean acute lesion score are plotted daily. (**b**) The total mean lesion score is compared, with the mean line indicated and proportion of animals that developed acute genital lesions shown with significance analysed via ANOVA with Tukey’s test to adjust for multiple comparisons, *p* < 0.001 (***).

After the two-dose immunisation schedule, the animals were challenged intravaginally three weeks later with HSV-2. All vaccine treatments significantly lowered the severity of acute disease compared to no vaccine. The gD DNA VLM formulations with or without chemokines CCL5 or VIT1 showed the most effective protection (Figure 3a). Thus, gD DNA + VLM alone showed 100% protection, with no observed lesions in the cohort or for total mean scores per individual (Figure 3a,b).

**Figure 4:**
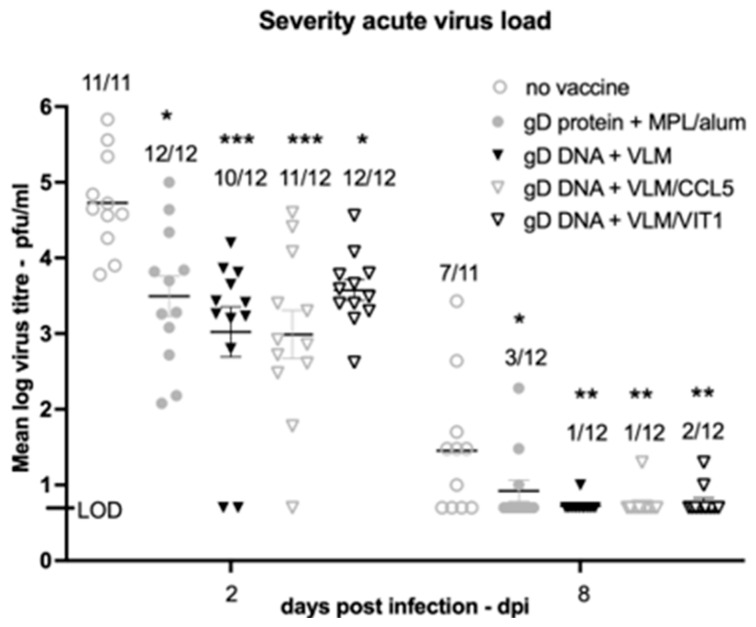
DNA immunisation with VLM vaccines protect against virus replication. The log_10_ geometric mean titre (GMT) was assayed for vaginal virus shed for each day post challenge inoculation, with the number of animals having detectable virus shown above each of the bars. LOD shows the limit of detection. The mean and standard deviation are indicated, on day 2 and day 8 pairwise comparisons to no vaccine are analysed, with *p* < 0.5 (*), <0.01 (**) and <0.001 (***).

Vaccines also significantly lowered vaginal virus replication (Figure 4), which correlated with the disease protection shown in Figure 3. There was approximately a 2-log reduction in virus shed with immunisations using gD DNA + VLM and gD DNA VLM + CCL5, compared to 1 log reduction by the gD protein formulation. By 8 days post virus challenge, the DNA formulation immunisations had reduced virus shedding to undetectable levels in almost all animals (Figure 4) with greatest reductions in virus titre with gD DNA + VLM.

### 3.4. Gene Immunisation Protective against Establishment HSV-2 Latency

**Figure 5:**
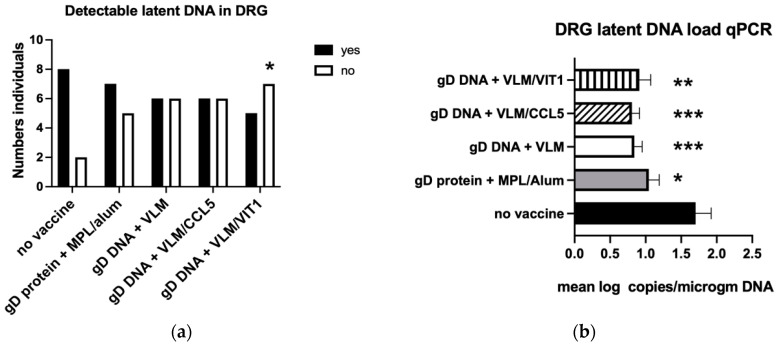

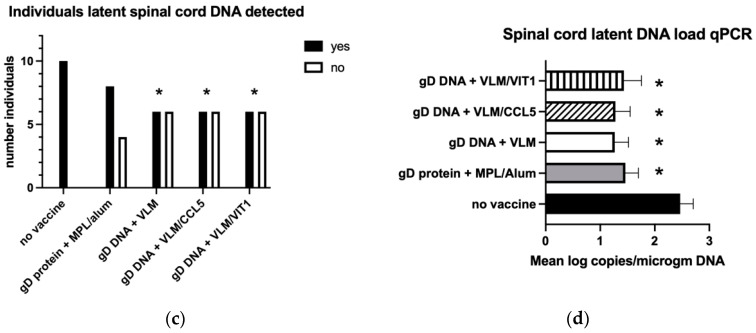
VLM immunisations protect against establishment of latency. Latent DNA was quantified by PCR showing in (**a**,**c**) detected in DRG or spinal cord, respectively, in individuals as analysed by one tailed chi square, (*) *p* < 0.05. In (**b**,**d**) the mean log copies/microgram of DNA measured are compared using one way ANOVA with Tukey’s test for multiple comparisons, (*) *p* < 0.05, (**) *p* < 0.01, (***) *p* < 0.001.

As the immunisations protected against acute disease and virus replication, the effects on establishment of latency were then examined. Only the VLM DNA immunisations significantly reduced the number of animals with detectable latent DNA in the DRG and overall reduced viral load (Figure 5a–d). In contrast, the gD protein vaccine did not significantly reduce detection of latent DNA in either the DRG or the spinal cord. Over half of the animals treated with gD DNA + VLM/VIT1 were protected, in that no detectable HSV-2 DNA was recovered in the DRG, compared to 80% detection in the no vaccine animals (Figure 5a). Analyses of the total mean DRG loads showed all vaccines significantly reduced HSV-2 DNA levels compared to no vaccine, with the gD DNA vaccines reducing the HSV-2 DNA by approximately 50% (Figure 5b). Analyses of latent DNA detected in the spinal cord showed mostly similar effects to the DRG. While all the vaccine treatments significantly reduced latent viral DNA load detected in the spinal cord, only the gD VLM DNA vaccine, and not the gD protein immunisation, had significantly reduced numbers of animals with detectable DNA in the spinal cord (Figure 5c,d).

### 3.5. Gene Immunisation Protective against Recurrent Disease

**Figure 6:**
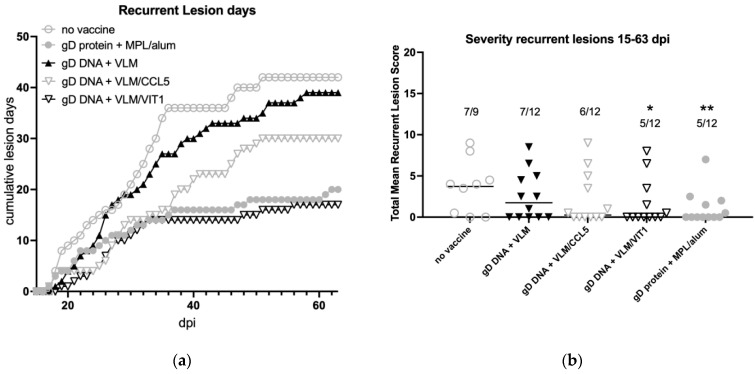
Immunisation with VLM and VIT protects against recurrent disease from day 21–63 following intravaginal challenge with HSV-2. The effect of immunisation on recurrent disease (**a**) and disease severity (**b**) were examined days 15–63 post intravaginal challenge with HSV-2 strain MS. The animals were evaluated daily for presence of recurrent lesions and scored. Comparisons of disease severity are shown in b with means and standard deviation indicated with pairwise comparisons to no vaccine analysed by Wilcoxon test, *p* < 0.05 (*) and < 0.01 (**).

The VLM vaccines protected efficiently against acute HSV-2 disease and as well as establishment of latency, therefore we examined the effects of the vaccine treatments on recurrences of disease and shedding from 15 to 63 days post infection. This showed that although the gD + VLM vaccine eliminated primary disease and showed some reductions in latency, it did not affect the development of recurrent lesions (Figure 6a). Further, there was only limited reductions shown with addition of the CCL5 gene to the gD+VLM immunised group. In contrast, immunisation including the VIT chemokine DNA in addition to the gD DNA + VLM reduced recurrent lesions that was similar to the gD protein formulation (Figure 6a). Both gD DNA + VLM/VIT and gD protein formulation reduced recurrent lesion days in those with disease by approximately 50%, while most of the animals were completely protected from any disease recurrences, 58% (7/12). Analyses of the total recurrent mean lesion score in Figure 6b shows only gD DNA + VLM/VIT and the gD protein immunisations significantly lowered total recurrent mean lesion scores.

### 3.6. Gene Immunisation Reduces Recurrent Virus Shedding and Lesions

**Figure 7:**
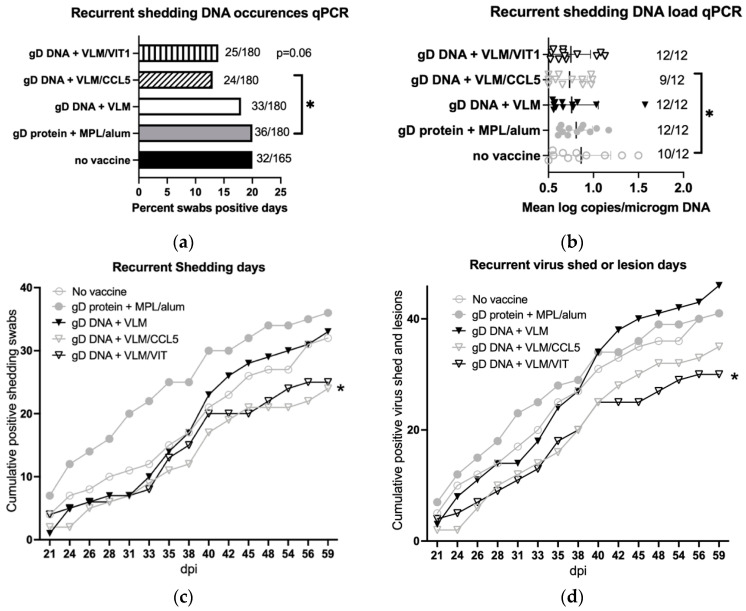
Immunisation with VLM vaccines with VIT show reductions in recurrent virus shedding in vaginal swabs or lesions from day 21–63. (**a**) Comparison of recurrent shedding occurrence as detected by quantitative DNA PCR above detection cut-off, one tailed chi square. (**b**) Recurrent shedding loads were compared between immunisations and no vaccine after HSV-2 challenge, as analysed in pairwise comparisons by Wilcoxon test, *p* < 0.05 (*). (**c**) Positive vaginal shedding swabs assayed by quantitative DNA PCR were recorded daily and cumulative events plotted. (**d**) Combined recurrent disease lesions and virus shedding events recorded cumulatively daily, *p* < 0.05 (*) two tailed chi square at end of study compared to no vaccine.

The effects of the vaccine treatments were also tested for reductions on recurrent virus shedding after day 20 post virus challenge. Immunisations with VLM and CCL5 reduced total virus recurrent shedding days by approximately 30%, with VIT showing a similar trend, while the gD protein showed no reductions (Figure 7a,b). Interestingly, although the gD protein immunisation had protected against recurrent disease, it appeared to increase daily virus shedding in comparison to the no vaccine control group (Figure 7c). In contrast, daily virus shedding was reduced only after immunisations with the gD DNA + VLM with chemokines VIT or CCL5 (Figure 7c). In combined analyses of cumulative daily totals of any recurrence, lesions or virus shedding, there was only significant reductions by gD DNA + VLM/VIT compared to no vaccine or gD protein + MPL/alum (Figure 7d). As summarised in Table 1, durable performance on both recurrent lesion days and reduced virus shedding as well as acute primary disease, was only shown by gD DNA + VLM and VIT immunisation.

## 4. Discussion

To control herpesvirus disease requires both inhibition of acute and recurrent infections. Clinical evaluation to control HSV-2 using monovalent protein vaccines even with potent adjuvants, have not been able to limit HSV-2 disease [8]. However, some efficacy against HSV-1 was shown, at least in part by antibodies, suggesting control should be possible [11,12]. Improving efficacy may require addition of more HSV proteins in multivalent vaccines. Approaches using whole virus with gene deletions address this, but raise the risk of recombination with latent virus or new infections, potential off target effects, as well as increasing co-morbidities [46,47].

In this study we have investigated the use of a novel stabilised virus entry complex and immunomodulator. This mimics the natural conformation of the virus entry complex, but eliminates the risk from using whole virus or virus vectors. The results shown here demonstrate efficient and durable control of the virus and related disease through these novel mechanisms. The VLM formulation gave complete protection from acute disease, reduced virus replication and latency, while addition of VIT eliminated disease recurrence in most animals and reduced vaginal virus shedding. The stabilized fusion conformation may elicit conformational antibodies to control virus infection, while VIT may stimulate processing linear epitopes for MHC presentation for cellular immunity to control recurrence from latently infected cells.

The first approach using VLM was designed to mimic the virus entry complex as presented naturally in membranes as antigens in the fusion complex [13,42]. The virus entry complex is composed of four membrane glycoproteins necessary and sufficient to mediate cell fusion by in vitro cellular assays [42,43,48]. Cell fusion mediated by herpesviruses is a first step for infection [14]. HSV encodes four essential glycoproteins, mediating cell fusion in gene transfections in vitro [14]. These sequentially interact and include the immunogen gD, as well as the fusogen gB and fusion regulators the gH/gL complex [14,45,49,50,51]. To induce an effective immune response, the host should be exposed to prefusion conformations in order to block roles of cell fusion in infection.

Effective SARS-CoV-2 vaccines, have targeted the spike stabilised prefusion structure [52,53]. Reducing cell fusion also correlates with decreases in pathogenic effects [54] as for herpesvirus spread within the host [13,55,56]. A pre-fusion structure of the HSV-1 fusogen gB utilised a single SNP in domain III, which reduced the ability of the hinge domain to undergo conformational change to trigger cell fusion [45], while a single SNP in domain I was modelled to stabilise the structural conformation mimicking interactions between fusion loop adjacent region in a related herpesvirus, HHV-6A [34]. This domain I SNP designed here for HSV-2 gB was able to mediate cell fusion in the in vitro cell fusion assays when co-expressed with gD, gH and gL, but was significantly reduced compared to cell fusion mediated by the domain III SNP in gB. This VLM prefusion stabilisation may participate in its efficacy in inducing neutralising antibodies which appear so effective in inhibiting all symptoms of acute infection in the guinea pig model.

Members of the virus entry complex, gD, gB, gH and gL have been evaluated previously as vaccines, as antigens rather than recreated stabilised membrane expressed virus entry complexes as shown here. For example, using the same guinea pig model, polypeptide vaccines comprising combinations of truncated gD; truncated gD + gB + gH/gL; or gB + gH/gL raised effective immunity, but none of these vaccines were more effective than gD alone [57]. In these protein vaccines, the conformational epitopes of the fusion complex would not be created and the antigen is secreted rather than membrane bound as in the natural virus or virus infected cells. Moreover, clinical trial evaluations of such truncated and secreted gD showed only partial protection for women to HSV-1 and no protection from HSV-2 [8,12].

Our second approach, addresses cellular immunity which may control recurrences via modulation of chemokine recruitment of T cell or antigen presentation populations. We investigated chemoattraction to enhance this process by modulating specific cellular subsets using CCL5 and VIT. We identified and utilised the novel chemokine modulator, VIT, in the immunisation together with VLM formulations. This resulted in decreased recurrent disease and eliminated recurrent lesions in most of the animals while also reducing the days with identified secreted virus. While all the vaccines lowered the latent HSV DNA load in the DRG and the spinal cord, only gD VLM/VIT significantly reduced the number of individuals with detectable latent DNA, with complete protection for 58%. Moreover, only gD VLM with VIT but not VLM on its own or gD protein/adjuvant vaccines reduced recurrences both by recrudescence (lesions) and virus reactivation (virus genital secretion). VIT is a human gene derived from an endogenous archaic virus genome, integrated in the human host, iciHHV6A [30]. Like other endogenous virus genomes these are now humanised genes [58]. We identified a novel spliced variant, VIT, defining a new molecular adjuvant which could control cellular immunity. VIT retains the N-terminal binding domain to multiple chemokine receptors but is lacking the original chemokine signaling domain, therefore it can act as an inhibitor. This novel spliced cDNA now encodes a short hydrophobic C-terminal 8 amino acid with tryptophan phenylalanine similar to other stabilised lipid associating peptides [59,60]. The human chemokine binding region has been previously characterised and includes efficient binding to CCR5, such as human CCL5, but with increased affinity [28,61]. CCR5 can be expressed on activated T lymphocyte populations. These could be helper or cytotoxic T effector T cells, and T regulatory subsets, or on antigen presenting cells such as monocytic/macrophage cells or dendritic cells [24,62]. Therefore, targeting CCR5 by co-expression with CCL5 in some contexts could be immune stimulatory but in other settings, could be suppressive. Indeed, as shown previously in the murine model, gD plus CCL5 showed some effectiveness [20], and this is shown here in the guinea pig model.

In contrast to CCL5, the VIT molecule could target CCR5 as well as CCR4, CCR6 and CCR8, which are on multiple T regulatory, T-Reg, subsets [22,63]. Therefore, a potential mechanism of action for VIT is to block recruitment of T regulatory subsets, to a site of immunisation, and thereby increasing an immune stimulatory signal. This appears to enhance a memory cellular response, since the effect on virus shedding was most durable with VIT over CCL5. Evidence for this adjuvant design is the co-immunisation of the gD protein with the actual agonists, human chemokine ligands for CCR4, CCR5, CCR6 and CCR8, our novel CIR formulation. Indeed, this group of guinea pigs with CIR, induced no HSV-1 specific antibodies, like the no vaccine controls. Moreover, there was no protection from virus challenge (no vaccine vs. gD + MPL/alum mean difference log titres 1.23 [95% CI 0.458–2.01], *p* = 0.002 compared to no vaccine vs. gD + CIR, 0.384 [95% CI −0.392–1.16], *p* = 0.42, no significant difference), despite immunisation with the powerful immunogen gD, that on its own induce antibodies and protects from initial infection in this model [39]. This supports the interpretation that VIT can redirect the T regulatory cells by blocking their recruitment via their chemokine receptors. Interestingly, recent report indicates autoantibodies to select chemokines can support favourable outcomes to disease, including COVID and HIV, and include inhibition of CCR4 and CCR6 as shown here by VIT, through a different mechanism [64]. The actions of VIT and its converse CIR, are both human specific, therefore although these were effective in guinea pigs, with conserved receptors, they should increase in humans.

The ability to use defined genes to deconstruct the virus, and to transiently stimulate the immune response by VIT action via chemokine inhibitor pathway as shown here using a DNA modality, offers a highly effective and potentially safer approach. Applications to RNA could also be employed, but the attractions of thermostability that DNA offers combined with defined and efficient single fermentation manufacturing for scaling offers advantages for utility in many settings.

Immunisation of the VLM + VIT DNA formulation protected against acute infection, latency establishment, and recurrent disease, with reduction in virus shedding, while in comparison the adjuvanted gD protein vaccine reduced recurrence but had no effect on virus shedding. In fact, adjuvanted gD protein immunisation appeared to increase recurrent HSV-2 virus secretion, trends seen in other animal models, but not for HSV-1 [33,39,65]. Effects of cell membrane TLR4 sensing of these adjuvant lipids [19], may differ from the endosomal TLR7, TLR9 [18] and secreted chemokine regulation shown here. Control of viral shedding is crucial to control of HSV as shed virus accounts for most transmission, and has evidence as appropriate surrogate for disease since on the causal pathway [7]. Overall, clearly novel VLM DNA vaccine is highly efficient completely protecting all against acute HSV-2, but does not protect from recurrences. VLM plus novel VIT DNA vaccine further protects most from HSV-2 recurrent disease and latency, plus reduces virus reactivation of vaginal secretion, supporting further clinical evaluation.

## 5. Patents

Virothera Ltd. has filed for patent protection for the aspects of the VLM, VIT and CIR vaccines described and their applications.

## Figures and Tables

**Table 1:** Vaccine efficacy protecting from acute or recurrent HSV-2 disease or infection.

Conditions ^1^ >50% Protection	No Vaccine	gD Protein + MPL/Alum	gD DNA + VLM	gD DNA + VLM/CCL5	gD DNA + VLM/VIT
Prevent acute disease	− (1/12)	75% (9/12)	100% (12/12)	100% (12/12)	75% (9/12)
Prevent virus replication	− (4/11)	75% (9/12)	92% (11/12)	92% (11/12)	83% (10/12)
Reduce latency load	−	+	+	+	+
Reduce latency detection DRG	−	−	−	−	+
Reduce recurrent disease	− (2/9)	58% (7/12)	− (5/12)	− (6/12)	58% (7/12)
Reduce recurrent virus shed	−	−	−	+	+/−
Reduce recurrent virus and disease	−	−	−	−	+
Overall	−	+	+	++	+++

^1^ Recurrence effects 2 months post-infection challenge, 2 immunisations.

## Data Availability

Sequences reported here are available at NCBI as cited.

## Supplementary Materials

The following supporting information can be downloaded at https://www.mdpi.com/article/10.3390/v14112317/s1, Figure S1: VIT splicing expressed in human cells, Figure S2: VIT encoded cDNA, Figure S3: RNA expression of VLM and VIT genes in human cells.
