# Supplementary material for: Immunisation Using Novel DNA Vaccine Encoding Virus Membrane Fusion Complex and Chemokine Genes Shows High Protection from HSV-2
Source: Viruses. 2022 Oct 22;14(11):2317. doi: 10.3390/v14112317 (PMC9698128; doi:10.3390/v14112317)
Supplement: Supplementary file 1 [file viruses-14-02317-s001.zip › viruses-1974972-supplementary.pdf]

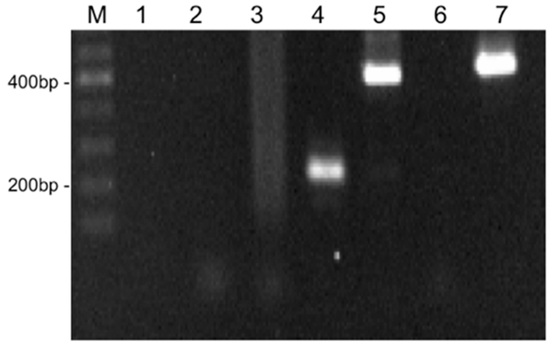

Figure S1. VIT splicing expressed in human cells. Cellular expression of iciU83A is spliced into a novel spliced cDNA iciU83A-N. The gene iciU83A was cloned into a plasmid expression construct pCMV6 and transfected into HEK293 cells. Lanes 1-3 are negative controls, reaction mix with no oligonucleotide primers, reaction mix with oligonucleotide primers, reaction mix with oligonucleotide primers and water only template. Lanes 4-7 are one step RT-PCR reactions of total RNA extracted from the transfected cells primed using primers from the plasmid vector, pCMV6neo (also with primers amplifying the iciU83A gene, not shown). Lanes 5 and 7 are untreated with DNase and show the residual DNA from transfection. Lane 4 and 6 are DNase treated. Lanes 4 and 5 include reverse transcriptase. Lane 5 shows the full-length DNA and Lane 4 shows the expressed spliced cDNA product, iciU83A-N, the virokine immune therapeutic tested here, VIT.

(a)

atg tcc att cgg ctt ttt att gg**t** **ttt** **ttt** **t**at acg gca tat att ggt atg gct atc gga
M S I R L F I G F F Y T A Y I G M A I G
ttt ata tgt agt tcc ccc gat gcg gag ctg ttt tcc gaa aaa tca cgt att tcg tct tct
F I C S S P D A E L F S E K S R I S S S
gtc ttg tta gga tgt ttg ttg tgt tgc atg gat tgg tcc gct gcc g**ta** **cc**c gtc tgg ttt
V L L G C L L C C M D W S A A V P V **W** **F**
gga gca ggg ctc gat gtg **tga**
G **A** **G** **L** **D** **V** *

(b)

... tgg tcc gct gcc g**ta** **cc**c gtc tga ...... W S A A V P V

***** **...**

Figure S2. VIT encoded cDNA. The cDNA sequence is shown of novel spliced transcript from human integrated iciHHV-6A U83A transfected in human HEK293 cells. This shows in (**a**) the splicing operates through DR, direct repeat, TACC indicated, despite a 3′ proximal mutation, TGA-TGG. This non-synonymous SNP in the full-length gene transforms this spliced product, unlike circulating virus, into mutation of the original spliced stop codon shown in (**b**). Comparing the spliced product from circulating virus (**b**) to spliced product of humanised integrated gene (**a**), the integrated cDNA allows read

through of the original spliced stop codon encoding 8 further amino acids and removes the C-terminal signalling domain of the circulating virus gene.

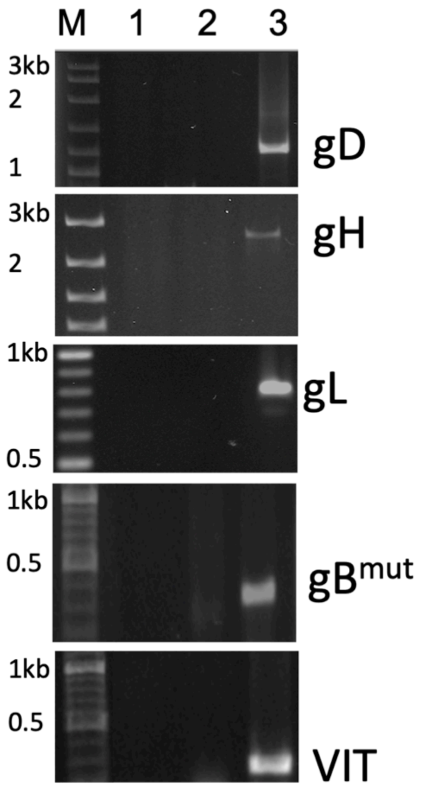

Figure S3. RNA expression of VLM and VIT genes in human cells. Cellular *in vitro* expression of VLM genes (gD, gB-mut, gH and gL) and VIT genes cloned into pCMV6 expression vector. The DNA plasmid formulation was transfected into HEK 293 cells and 48h post transfection total RNA was extracted then analysed by one-step RT-PCR using oligonucleotide primers specific for the genes as described in methods. The RNA was DNase treated, lanes 2, 3, and then reverse transcriptase treated, lane 3, or untreated, lane 2. Negative control in lane 1 is reagents without RNA. Lane M shows the DNA markers. Shown is representative of 3 assays, gB^{mut} indicates the gB gene with SNP mutation described in the text, VIT, expressed from the cDNA as shown in Figure S2.
